# Phytochemical analysis and green synthesis of silver nanoparticles using *Centella asiatica* leaf and stem extracts: An investigation of antibacterial activity

**DOI:** 10.1371/journal.pone.0321172

**Published:** 2025-09-25

**Authors:** Kamana Sharma, Manisha Bhusal, Akash Budha Magar, Ishwor Pathak, Lok Kumar Shrestha, Khaga Raj Sharma

**Affiliations:** 1 Central Department of Chemistry, Tribhuvan University, Kathmandu, Nepal; 2 Department of Chemistry, Amrit Campus, Tribhuvan University, Kathmandu, Nepal; 3 Research Center for Materials Nanoarchitectonics (MANA), National Institute for Materials Science (NIMS), Tsukuba, Ibaraki, Japan; Universitas Airlangga, INDONESIA

## Abstract

*Centella asiatica* (L.) Urban is a traditionally revered plant possessing several therapeutic applications. This research evaluated the phytochemical and antibacterial properties of *Centella asiatica*. In addition, silver nanoparticles were synthesized using the stem and leaf aqueous extracts. The presence of alkaloids, flavonoids, glycosides, terpenoids, and phenolics in plant extracts indicates their high medicinal value. Methanolic leaf extract showed a higher phenolic and flavonoid content of 43.73 ± 0.33 mg GAE/g and 19.76 ± 1.12 mg QE/g, respectively. It also contained the most antioxidant activity with the lowest DPPH inhibitory concentration (IC_50_) of 49.31 ± 1.48 µg/mL. Plant extracts and synthesized nanoparticles were active against gram-positive and gram-negative bacteria. Methanolic leaf extract displayed an MIC of 27.5 and MBC of 55 mg/mL against *S. aureus*. Synthesized nanoparticles were characterized using different spectroscopic techniques. UV-visible spectra of nanoparticles contained distinct absorption peaks resulting from surface plasmon resonance at 405 and 408 nm. The nanoparticles showed face-centered cubic crystallinity in powder X-ray Diffraction analysis. Fourier Transfer Infrared spectra suggested the possible involvement of organic functional groups in nanoparticle synthesis. Field Emission Scanning Electron Microscopy and Transmission Electron Microscopy analysis revealed the spherical shape with non-uniform size distribution. Mean particle sizes were 20 nm and 19 nm for leaf and stem extract synthesized nanoparticles*,* respectively*.* In conclusion, *Centella asiatica* is rich in important plant secondary metabolites and biological activities, and its aqueous extract synthesizes silver nanoparticles that show potential antibacterial activity.

## 1. Introduction

Plants provide a large number of compounds that can be used as environmentally friendly medicinal sources. The biome of Nepal is a natural pharmacy containing many therapeutically beneficial herbs due to its unique geographical features, diverse topography, and biodiversity [1,2]. Over 13,000 plant species are reported from Nepal, among which 2,500 have documented medicinal use [3]. Plants produce primary metabolites for growth and development, whereas secondary metabolites such as polyphenols, terpenes, steroids, and essential oils are produced for defense and other cellular processes [4]. These secondary metabolites serve as the basis for the development of novel medications. They can be used either in their native state or as semi-synthetic derivatives for the effective management of a wide range of human conditions, including cancer, hypercholesterolemia, inflammation, microbial infections, and tissue rejection in organ transplantation [4,5].

Active phytoconstituents isolated from the different parts of medicinal herbs contain minimal side effects [6]. Plant-derived antioxidants exert health benefits by reducing oxidative stress [7]. In addition to medicinal properties, medicinal herbs are being employed for the green synthesis of nanoparticles. The material dimensions of these nanoparticles give rise to special properties not available in the macroscale bulk material [8]. The scientific study of materials at the nanoscale has become more accessible through nanotechnology. Nowadays, researchers can monitor, assess, and work on nanoscale systems at the atomic and molecular levels. Nano-architectonics has arisen as a post-nanotech concept for the creation of functional materials using nanostructures [9]. In this regime, metal nanoparticles are exceptionally persistent, stable, and efficient heterogeneous catalysts with many applications [10]. Thus, prepared nanoparticles have shown promising antibacterial, antioxidant, and anticancer activities and drug-delivery potential [11].

The production of silver nanoparticles (AgNPs) using plant-mediated processes is currently gaining recognition as a unique and important discipline in nanotechnology due to its eco-friendliness [12]. AgNPs are employed in diverse sectors of electronics, optics, photography, textiles, catalysis, food industry, dentistry, and biomedical applications [13]. Silver is one of the frequently used elements in the manufacture of nanoparticles, and there is a long history of its use in antimicrobial treatments [14,15].

*Centella asiatica* (L.) Urban is a blooming herbaceous perennial plant, commonly known as Ghodtapre in Nepal. It is a member of the Apiaceae family. It is also the most ubiquitous species in the genus *Centella* with many medicinal properties [16]. This plant grows in moist and shady areas with an average height of 12–15 cm. The stem is striated, glabrous, and rooted from the nodes [17]. It has small, fan-shaped green leaves and is odorless, tasteless, or somewhat bitter. The flowers are white or pale purple to pink, and the fruit is small in size with oval in shape. The leaves and stems are often utilized for therapeutic purposes [18]. The plant contains various pentacyclic triterpenoids with pharmacological activity, including asiatic acid and asiaticoside [17]. It also contains brahmoside, centelloside, kaempferol, quercetin, rutin, catechin, naringin, and apigenin [19]. In traditional medicine, the plant is used for the treatment of dermatitis, arthritis, irritation, psychological disorders, seizures, diarrhea, leprosy, ulcerations, bronchitis, cough, ivory fever, stress, urinary tract infections, vision issues, and post-injury recovery [20]. It is used to alleviate dryness of the skin in individuals with diabetes [21]. Tribal people of Southern India use it to treat wounds, whereas the Hodi tribe from Bangladesh employs it for jaundice [22]. In Nepal, the people of the Dolpa district use it for toothache, indigestion, pneumonia, and dysentery [23]. The Newar community employs it as an antidote to poison [24].

The widespread ethnobotanical uses of *Centella asiatica* have garnered the attention of the scientific community, but there is a lack of comparative studies involving stem and leaf extract. In this contribution, the researchers have evaluated the phytochemical and antibacterial properties of *Centella asiatica* leaf and stem extracts and also reported a facile fabrication and characterization of silver nanoparticles using aqueous extracts of the leaf and stem of the plant.

## 2. Materials and methods

### 2.1. Materials

The leaves and stems of *C. asiatica* were collected from wards number 2, 6, and 7 of Musikot municipality, Gulmi, Nepal, in October 2022. The plant samples collected for the study are not considered an endangered species in Nepal. So collection and study of these plant samples becomes easy and does not require permission to work on them. The coordinates of the plant collection site are 28^○^ 07’ to 28^○^ 21’ North and 83^○^ 11’ to 83^○^ 24’ east. The altitude ranged from 992 m to 1108 m with a subtropical climate. The plant was identified by the National Herbarium and Plant Laboratories (NHPL), Godawari, Nepal, with voucher specimen KS-001 (KATH). The leaves and stems were thoroughly rinsed using purified water to eliminate contaminants, followed by chopping. The pieces were left to dry in the dark for 2 weeks, then ground to a smooth powder using an electric grinding device. The powder was stored in individual waterproof zip-locked plastic bags until further use. All reagents used in subsequent experiments were of analytical grade. Solvents (methanol, ethanol, ethyl acetate, hexane, dichloromethane (DCM), dimethyl sulphoxide) and other chemicals including gallic acid, Quercetin, DPPH, Folin-Ciocalteu reagent (FCR), aluminum chloride (AlCl_3_), potassium acetate (CH_3_COOK), sodium carbonate (Na_2_CO_3_) and (AgNO_3_) were purchased from Merck or ThermoFischer Scientific, USA. Neomycin, Muller Hinton Agar (MHA), and Muller Hinton Broth (MHB) were obtained from Hi-media, India. Bacterial strains (American Type Culture Collection (ATCC) of *Escherichia coli* ATCC 2591, *Klebsiella pneumoniae* ATCC 700603, *Staphylococcus aureus* ATCC 43300, and *Shigella sonnei* ATCC 25931) were obtained from the Central Department of Chemistry, Tribhuvan University, Kirtipur, Nepal.

### 2.2. Preparation of plant extract and phytochemical analysis

22 g each of powdered leaves was added to two conical flasks containing 220 mL each of methanol and hexane. Similarly, 29 g each of stem powder was dissolved in 290 mL each of methanol and hexane. The contents were left for maceration for 72 hours with continuous stirring. Methanol and hexane were used as solvents; polarity plays an important role in phytochemical extraction [25]. It was assumed that methanol would extract more polar phytochemicals and hexane would extract less polar ones. After maceration, the contents were filtered and the filtrates were evaporated on a water bath at 40–45 ºC to obtain crude extracts. Percentage yields were calculated as percentage yield = W_1_ (g)/ W_2_ (g) × 100. Where ‘W_1_’ and ‘W_2_’ represent dry weights of extract and sample, respectively. The crude extracts were tested qualitatively to detect the presence of secondary metabolites [26].

### 2.3. Estimation of total phenolic and flavonoid content

TPC and TFC were estimated using the Folin-Ciocalteu reagent technique and the aluminum chloride colorimetric techniques, respectively [27,28]. Standard solutions of gallic acid were prepared at concentrations of 100, 90, 80, 70, 60, 50, 40, 30, 20, and 10 µg/mL. Triplicates of gallic acid solutions (20 µL) and extract samples (20 µL, 5000 µg/mL) were added to the wells of a microtiter plate. Then, FCR solution (10%, 100 µL) and Na_2_CO_3_ (1M, 80 µL) were also added to each well. The microplate was placed in the dark for 30 minutes, and then the absorbance of reaction mixtures was measured at 765 nm with the help of a microplate photometer (Epoch 2, Biotek, Instruments, Inc., USA). TPC was calculated by using a standard gallic acid calibration curve.

For TFC, standard solutions of quercetin at different concentrations (10, 20, 30, 40, up to 100 µg/mL) were prepared in ethanol. Triplicates of quercetin solutions (10–100 µg/mL, 130 µL) and extract samples (20 µL, 5000 µg/mL) were added to the wells of another microtiter plate. The wells containing the extract samples received an additional 110 µL of distilled water. Then, AlCl_3_ (10%, 5 µL), CH_3_COOK (1 M, 5 µL), and ethanol (60 µL) were also added to each well. The plate was incubated in a dark place for 30 minutes. Then, absorbance was measured at 415 nm. TFC was calculated by using a quercetin calibration curve.

### 2.4. Antioxidant potential

The standard protocol for DPPH assay was used to evaluate the antioxidant activities of plant extracts [29]. Solutions of quercetin (40, 20, 10, 5, 2.5, 1.25 µg/mL) were prepared in methanol, and it was used as a positive control. Solutions of methanolic extracts (500, 250, 125, 62.5, 31.25, 15.625 µg/mL) and hexane extracts (2000, 1500, 1000, 500, 250, 125 µg/mL) were prepared in 50% DMSO (in water). Triplicates of quercetin solutions (100 µL) and plant extracts (100 µL) were added to the wells of a 96-well plate. Each well received a further 100 µL of 0.1 mM DPPH. After incubation in the dark for 30 minutes, the absorbances were measured at 517 nm. 50% DMSO was used as a negative control. Percentage inhibition was measured as percentage inhibition = (Ac – As)/Ac × 100. Where ‘Ac’ and ‘As’ represent absorbances of control and sample, respectively. The percentage inhibition versus concentration curve was employed to calculate IC_50_.

### 2.5. Antibacterial activity

The standard protocol of the agar well diffusion method was used for the antimicrobial assay of plant extracts [30]. A broth culture of test organisms was prepared in MHB and incubated for 12–14 hours. Then its turbidity was matched with McFarland’s standard (0.5). The dried MHA plates were carpet-cultured in Petri dishes using sterilized cotton buds dipped into the prepared bacterial inocula. Cavities (diameter = 6 mm) were prepared in the Petri dishes using a sterilized borer and appropriately labeled. Aliquots of 100 µL plant extract solutions (100 mg/mL), positive control Neomycin (1 mg/mL), and 50% DMSO (negative control) were added to the wells of the well plate. After incubation at 37 ºC for 24 h, the formation of clear zones of inhibition (ZOI) around the wells representing the bacterial growth suppression was measured.

### 2.6. Minimum inhibitory concentration (MIC) and minimum bactericidal concentration (MBC)

To determine MIC and MBC, the broth microdilution method was carried out following the protocols from the Clinical and Laboratory Standards Institute (CLSI) [31]. Two-fold serial dilution of crude methanolic leaf extract (220 mg/mL) was performed directly in sterilized microtiter plates containing MHB. The culture of bacteria was compared with a 0.5 McFarland standard solution and further maintained at a concentration of 10^6^ CFU/mL by diluting 1:100 in nutrient broth media. Each well received 5 µL of this bacterial suspension. Chloramphenicol (1 mg/mL) was used as a positive control and further serially diluted to two-fold concentrations. Following incubation at 37 ºC for 24 h, Resazurin pigment (0.003%) was added to all the cavities, and the plate was returned to the incubator for about 2–3 hours. Wells with inhibited bacterial growth remained blue while those containing bacterial colonies turned pink. The minimal extract concentration inhibiting bacterial development was interpreted as MIC. MBC was ascertained by spreading the contents of the wells on Nutrient Agar plates, followed by incubation overnight at 37 ºC.

### 2.7. Synthesis and characterization of AgNPs

Standard protocols were adopted for the synthesis of AgNPs from leaf and stem parts of *C. asiatica* [32]. The powdered plant sample (5 g) was mixed with distilled water (100 mL) and gently heated for about 15–20 minutes at 60 ºC with continuous stirring on a magnetic hot plate stirrer. The solution was then cooled, and filtered, and the resulting aqueous extract was treated with AgNO_3_ solution (1 mM) at different ratios (1:1, 1:4, 1:8, 1:9, and 1:10). Nanoparticle synthesis was most successful in 1:9 mixture for stem and 1:10 mixture for leaf extract as indicated by a sharp color transition from yellowish to reddish brown. After the completion of the reaction, the mixture was centrifuged for 30 minutes at 9,000 rpm to separate the synthesized nanoparticles. These were then washed using distilled water, dried in a desiccator, and placed in a refrigerator for later use.

A UV-visible spectrophotometer (Endress plus Hauser, Specord 200) was used to monitor the plant-mediated synthesis of silver nanoparticles in each of the reaction mixtures by scanning at a medium scan rate in the wavelength range of 300–600 nm. Distilled water was used as a reference to perform baseline correction of the spectrophotometer. Fourier-transform infrared (FTIR) spectra were obtained using Shimadzu IR Tracer-100 to identify the phytochemicals contributing to the production of AgNPs. Powder X-ray diffraction (XRD) spectra were collected on a Bruker D2 phaser diffractometer using CuKα (λ = 1.54 A^○^) radiation operating at 30 kV voltage in the 2θ range of 10º to 80º to assess the crystallinity. Surface morphology and structure were studied using scanning electron microscopy (SEM) and transmission electron microscopy (TEM). SEM images were recorded on an S-4800, Hitachi Co., Ltd., Tokyo, Japan, operated at an acceleration voltage of 10 kV (10 µA current). TEM and high-resolution TEM (HR-TEM) images were recorded on a JEM 2100F transmission electron microscope operated at 200 kV (JEOL Ltd., Tokyo, Japan). Energy dispersive X-ray (JEM-2100 plus) was used to study the elemental composition.

### 2.8. Antibacterial activity of AgNPs

The antibacterial properties of synthesized AgNPs were assessed by following the standard disc diffusion method [33]. Bacterial inocula (0.5 McFarland standards) were prepared overnight in MHB media at room temperature. Then, it was evenly applied to MHA plates using sterile cotton swabs, followed by air-drying for 15 minutes in a laminar airflow hood. Discs prepared by cutting Whatman filter paper were autoclaved and impregnated with AgNPs (15 mg/mL), aqueous extracts (15 mg/mL), positive control neomycin (1 mg/mL), and negative control distilled water. The discs were then placed on agar plates in the marked spots. Following a 24-hour incubation period at 37 °C, the ZOI encircling each dish on the Petri plates was measured.

### 2.9. Statistical analysis

All the tests were performed in triplicate, and results are presented as mean ± standard deviations. Values were compared with one-way ANOVA followed by Tukey’s test. Values with p < 0.05 were considered significantly different. Microsoft Excel 2019, GraphPad Prism 10.4.1, and Origin 2025 software were used for calculations.

## 3. Results and discussion

### 3.1. Percentage yield

Stem methanolic extract of the plant showed the highest percentage yield with 18.83%, followed by leaf methanolic extract with 14.72%. Hexane extracts displayed comparatively lower percentage yields with 1.05% for leaf and 1.04% for stem.

### 3.2. Phytochemical analysis

The phytochemical screening results of *C. asiatica* stem and leaf extracts are provided in Table 1. The phytochemical composition varied between hexane and methanolic solvent extracts. Methanolic extracts contain flavonoids, terpenoids, phenolics, glycosides, steroids, and proteins. Hexane, a nonpolar solvent, failed to extract many phytochemicals, which are generally polar. Only alkaloids and saponins were detected in the hexane extract. Similar findings are reported by Saranya et al. [34]. Slight variations in phytochemical compositions may be observed depending on the natural habitat and altitude of the sample collection site, climatic conditions, season of collection, and environmental conditions [35]. The phytochemicals present in the plant extract act as scaffolds for the synthesis of silver nanoparticles. They are involved in the reduction and stabilization of nanoparticles [36]. In addition, phytochemicals are responsible for various medicinal properties in plants [37]. Plant secondary metabolites, including phenolics, flavonoids, and carotenoids, are renowned for their antioxidant activities [38]. Phytochemicals are known to suppress or inhibit cancer growth [39]. Flavonoids contain the ability to repair wounds [40]. Plant secondary metabolites are responsible for the antibacterial, anti-inflammatory, antidiabetic, and antiurease activities of plants [41].

**Table 1:** Phytochemical analysis of *C. asiatica* methanol and hexane extracts.

Phytochemicals	Test	Leaf extract		Stem extract	
		Methanol	Hexane	Methanol	Hexane
Alkaloids	Wagner’s, Hager’s	–	+	–	+
Flavonoids	Lead acetate, NaOH	+	–	+	–
Carbohydrates	Molisch	–	–	–	–
Proteins	Xanthoproteic	+	–	+	–
Saponins	Froth	–	+	–	+
Glycosides	Fehling	+	–	+	–
Terpenoids	Chloroform	+	–	+	–
Tannin/phenolic	5% Ferric chloride	+	–	+	–
Steroids	Salkowski’s	+	–	+	–

+ Presence and – absence.

### 3.3. Total phenolic and flavonoid content

*C. asiatica* showed significant quantities of TPC and TFC in its leaf and stem extracts (Table 2). Both the phenolics and flavonoids were recorded at higher concentrations in the methanolic extract compared to the hexane extract. Leaf methanolic extract displayed the highest amounts of phenolics (43.73 ± 0.33 mg GAE/g) and flavonoids (19.76 ± 1.12 mg QE/g). It was followed by stem methanolic extract (TPC = 29.52 ± 1.03 mg GAE/g, TFC = 11.05 ± 0.50 mg QE/g). Hexane extracts of stem (TPC = 6.20 ± 0.76 mg GAE/g, TFC = 2.76 ± 0.10 mg QE/g) and leaf (TPC = 2.67 ± 0.83 mg GAE/g, TFC = 9.10 ± 0.30 mg QE/g) showed comparatively lower TPC and TFC. Phenolic and flavonoid contents are influenced by the composition and polarity of solvents [42]. Rashid et al. have reported comparatively higher TPC (88.62 ± 0.41 mg GAE/g) and TFC (211.34 ± 0.10 mg QE/g) in the methanolic extract of the same plant species [43]. Slight variations in TPC and TFC may be observed due to geographical diversity, presence of an interfering substance, polarity of solvents, sample-to-sample variation of plants, differences in analytical assay methods, or selection of standard and protocol. Phenolics and flavonoids are reported for antioxidant, anticancer, antibacterial, and cardioprotective effects [44]. High concentrations of these compounds in *Centella asiatica* indicate its medicinal value.

**Table 2:** TPC and TFC in different extracts of *C. asiatica.*

Plant extracts	TPC (mg GAE/g)	TFC (mg QE/g)
CLM	43.73 ± 0.33^a^	19.76 ± 1.12^b^
CLH	2.67 ± 0.83 ^a^	9.10 ± 0.30 ^b^
CSM	29.52 ± 1.03 ^a^	11.05 ± 0.50 ^b^
CSH	6.20 ± 0.76 ^a^	2.76 ± 0.10 ^b^

CLM: *Centella* leaf methanolic extract, CLH: *Centella* leaf hexane extract, CSM: *Centella* stem methanolic extract, CSH: *Centella* stem hexane extract. Values followed by the same letter in the same column are significantly different from each other at p < 0.05.

### 3.4. Antioxidant potential

**Fig 1:**
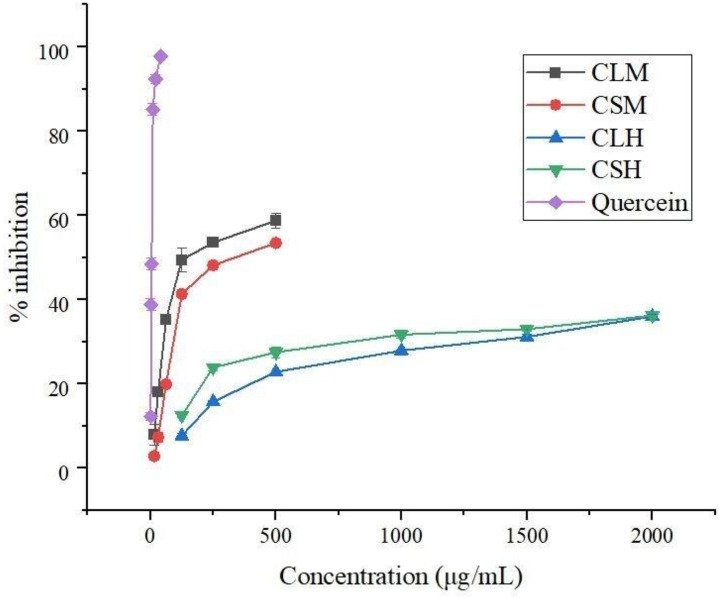
Percentage inhibition vs. concentration graphs obtained from DPPH assay. Here, CLM: *Centella* leaf methanolic extract, CSM: *Centella* stem methanolic extract, CLH: *Centella* leaf hexane extract, CSH: *Centella* stem hexane extract.

**Fig 2:**
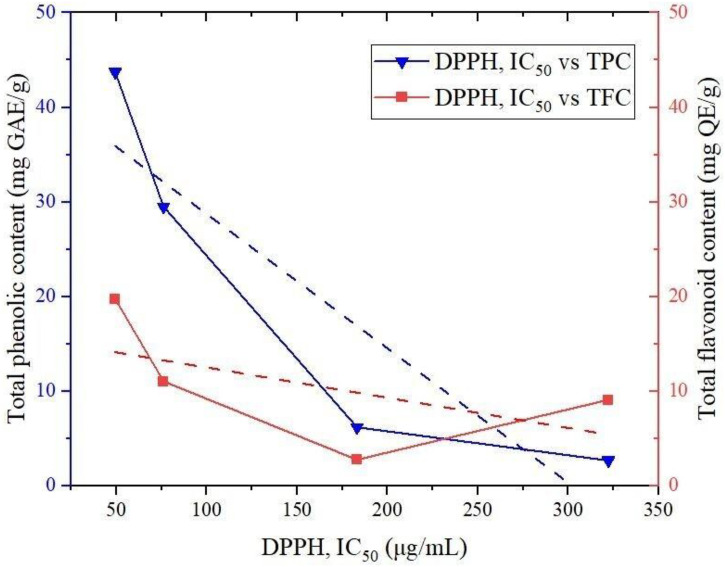
Correlations of antioxidant activity (IC_50_) with TPC and TFC.

The antioxidant activities of *C. asiatica* extracts were measured based on their capacity to neutralize DPPH free radicals. The calculated IC_50_ values shown in Table 3 reveal the excellent antioxidant properties of crude methanol extracts compared to hexane extracts. The IC_50_ value is inversely proportional to the antioxidant activity. Hence, a low IC_50_ value represents high activity. Leaf methanolic extract showed an IC_50_ of 49.31 ± 1.48 µg/mL, while the IC_50_ value of 76.08 ± 0.49 µg/mL was reported for stem methanolic extract. Hexane extracts of leaf and stem presented IC_50_ values of 322.3 ± 0.53 and 183.1 ± 0.56 µg/mL, respectively. Concentration dependency was observed for the antioxidant activity (Fig 1). With an increase in the concentration of extract, the inhibition percentage also increased significantly. Rashid et al. have reported an IC_50_ value of 24.19 ± 0.52 µg/mL for the methanolic extract of the same plant species [43]. IC_50_ values showed negative correlations with TPC (Pearson’s r = −0.902) and TFC (Pearson’s r = −0.565), as shown in Fig 2. Thus, antioxidant activity depends upon the phenolic and flavonoid content. This property of the plant reduces oxidative stress and prevents damage to cells, proteins, DNA, and molecules [45].

**Table 3:** IC_50_ (DPPH) values of *C. asiatica* extracts and standard quercetin.

Plant extracts	IC_50_ (µg/mL)
CLM	49.31 ± 1.48^*^
CLH	322.3 ± 0.53^*^
CSM	76.08 ± 0.49^*^
CSH	183.1 ± 0.56^*^
Quercetin	3.94 ± 1.43^*^

Values followed by ‘*’ are significantly different from each other at p < 0.05.

### 3.5. Antimicrobial activity

**Fig 3:**
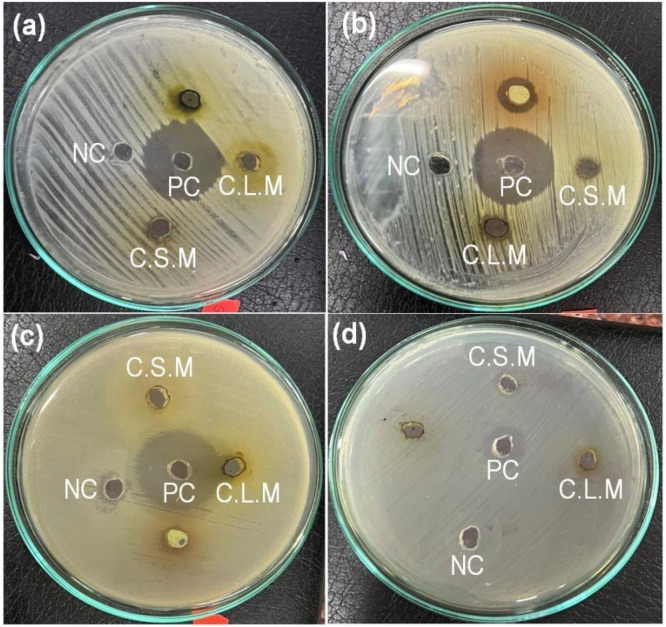
Photographs of ZOI formation against the test bacteria. Here, (a) *S. aureus*, (b) *S. sonnei*, (c) *K. pneumoniae*, and (d) *E. coli.*

Preliminary phytochemical analysis and thin-layer chromatography (TLC) showed that methanolic extracts of *C. asiatica* are richer in phytoconstituents than hexane extracts. The methanolic extract also contained significantly higher TPC, TFC, and antioxidant activity. Higher activity in the methanol extract is due to its high polarity, which is more effective in extracting relatively polar phenolics and flavonoids. A previous study by Bhusal et al. has also reported high activity in the methanol extract compared to the hexane extract [42]. Thus, preliminary screening of antibacterial activity was performed on the methanolic extracts. ZOI recorded against various bacteria is presented in Table 4 and Fig 3.

**Table 4:** ZOI against tested bacteria for the methanolic extracts of *C. asiatica* leaf and stem.

Bacteria	Zones of inhibition (mm)		
**CLM**	**CSM**	**Neomycin**
*S. aureus*	10	8	25
*S. sonnei*	10	8	25
*K. pneumoniae*	8	0	25
*E. coli*	0	0	12

Methanolic extracts of the stem and leaf showed significant activity towards gram-positive *Staphylococcus aureus* and gram-negative *Shigella sonnei.* Leaf extract was comparatively more effective as it showed a ZOI of 10 mm against both bacteria, whereas the ZOI for stem extract was recorded as 8 mm. Only leaf extract was active against *K. pneumoniae,* whereas *E. coli* was unaffected by both extracts. The control drug (Neomycin) exhibited stronger antibacterial action than the plant extracts. The ZOIs displayed by extracts in the present study are slightly lower than the ZOIs of 17 and 16 mm reported for the stem methanolic extract against *S. aureus* [46]. Similarly, DCM: MeOH extract (250 mg/mL) of the *C. asiatica* exhibited ZOIs of 9.67 ± 0.33 against *S. aureus* and 9.00 ± 0.58 mm against *S. sonnei* [33]. The observed antibacterial activity justifies the traditional uses of the plant against infectious diseases and wounds.

### 3.6. Minimum inhibitory concentration (MIC) and minimum bactericidal concentration (MBC)

**Fig 4:**
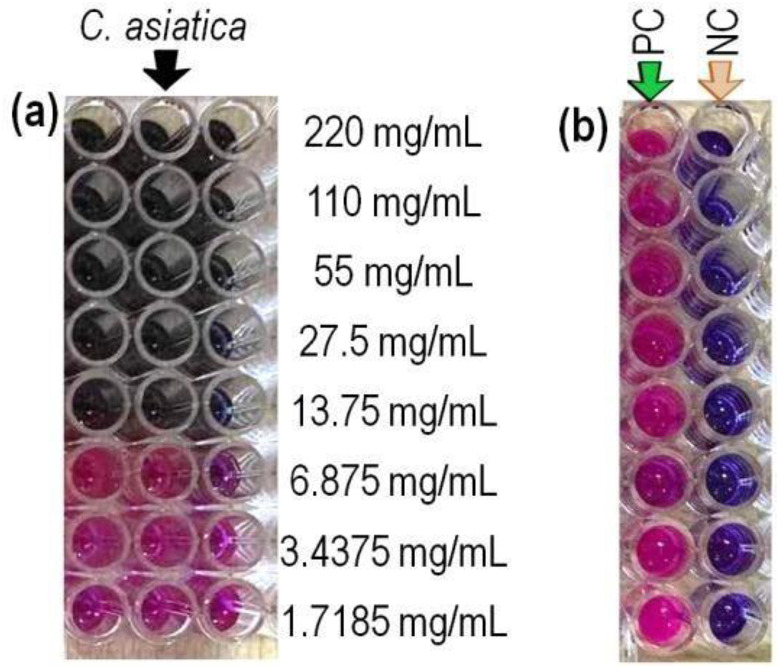
Determination of MIC and MBC. (a) Results of MIC and MBC for *C. asiatica* leaf methanolic extract against *S. aureus*. (b) The corresponding MIC and MBC for the standard positive control (PC) and negative control (NC).

Based on the findings of the agar well diffusion assay, leaf methanolic extract was selected for the determination of MIC and MBC against *S. aureus*. It displayed MIC of 27.5 mg/mL and MBC of 55 mg/mL (Table 5). These values are very close to 26.04 ± 5.21 mg/mL and 52.10 ± 10.4 mg/mL reported by Sieberi et al. for the DCM: MeOH extract of *C. asiatica* from Nairobi, Kenya [33]. The MIC and MBC shown by the methanolic leaf extract is shown in Fig 4. Thus, the methanolic extract of *C. asiatica* contains significant antibacterial activity.

**Table 5:** MIC and MBC of leaf methanolic extract against *S. aureus.*

Bacteria	Leaf Methanolic Extract	
	MIC (mg/mL)	MBC (mg/mL)
*S. aureus*	27.5	55

### 3.7. Synthesis and characterization of AgNPs

**Fig 5:**
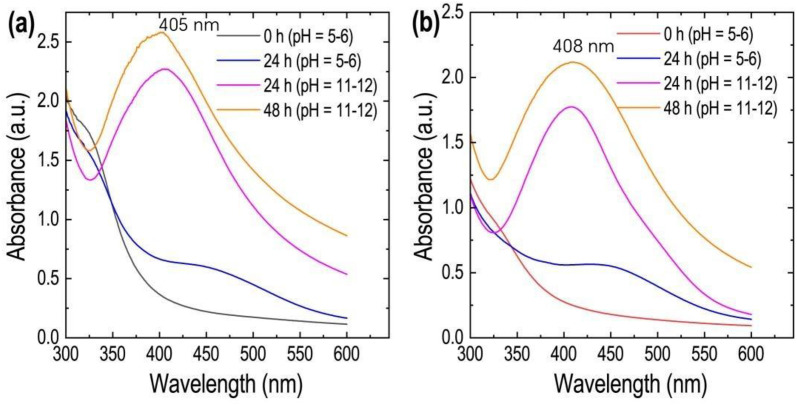
UV-visible spectra of AgNPs synthesized using *C. asiatica* (a) leaf extract and (b) stem extract at different time intervals.

The addition of leaf and stem extracts to AgNO_3_ solution in 1:10 and 1:9 ratios, respectively, followed by constant stirring over a magnetic stirrer, led to a color transition from yellowish to reddish, indicating the formation of AgNPs. UV-visible spectra were collected at different intervals (0 hours and 24 hours without the addition of reagents, and 24 hours and 48 hours after pH adjustment) to confirm the synthesis of AgNPs. The spectra are provided in Fig 5. Surface plasmon resonance (SPR) peaks were detected around 405 and 408 nm for silver nanoparticles synthesized using aqueous extracts of leaves and stems, respectively. These peak values are very similar to the SPR peak value of 409 nm reported for *Persicaria perfoliata-*synthesized silver nanoparticles [47]. The pH of the reaction medium was adjusted by adding the required amounts of HCl and NaOH. The formation of silver nanoparticles took place around the pH range of 11–12.

**Fig 6:**
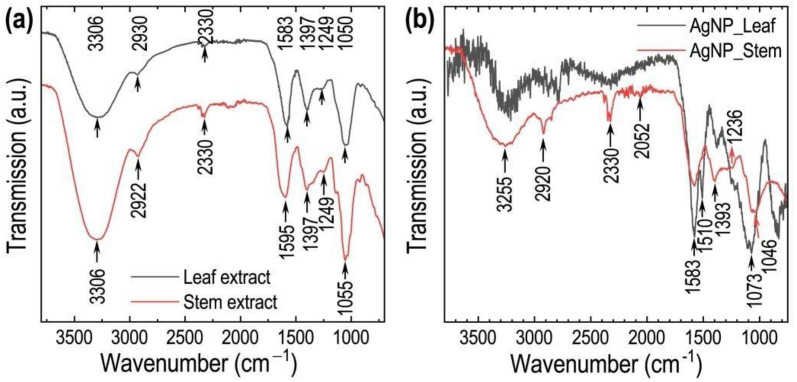
(a) FTIR spectra of *C. asiatica* leaf and stem extracts, and (b) FTIR spectra of AgNPs prepared using *C. asiatica* leaf and stem extracts.

The FTIR spectra of extracts and AgNPs were measured, and the peak positions were compared to ascertain the functional groups associated with capping or stabilizing activity (Fig 6). Stem aqueous extract displayed peaks at 3306, 2922, 2330, 1595, 1397, 1249, and 1055 cm^-1^. These peaks have shifted to 3255. 2920, 2330, 2052, 1236, and 1046 cm^-1^ in the spectrum of silver nanoparticles synthesized using the stem aqueous extract. Similarly, the FTIR spectrum of *C. asiatica* leaf extract showed absorption peaks at 3306, 2930, 2330, 1583, 1397, 1249, and 1050 cm^-1,^ while the spectrum of AgNPs synthesized using leaf aqueous extract showed peaks at 1583, 1510, and 1073 cm^-1^. The shifting of the broadband at 3306 cm^-1^ to 3255 cm^-1^ is caused by the interaction of polyphenolic -OH groups with AgNPs. The vibrations from 1073 to 1050 cm^-1^ are related to the C-O stretching of flavones [13]. The peak at 2330 cm^-1^ in the extract indicates the presence of nitrile groups [48]. Peaks in the region 1583–1510 cm^-1^ are due to C = C bond stretching, and the peak at 1397 cm^-1^ represents C-N groups [49]. The band at 1249 cm^-1^ is caused by stretching vibrations of C-O-C subunits in phenols. The C-H stretching of aliphatic groups is indicated by the band at 2930 cm^-1^ [50]. Functional groups such as hydroxyl (-OH), amide (-CONH), and carbonyl (C = O) in *C. asiatica* leaf extract play a vital role in the depletion of silver ions to form AgNPs [50].

**Fig 7:**
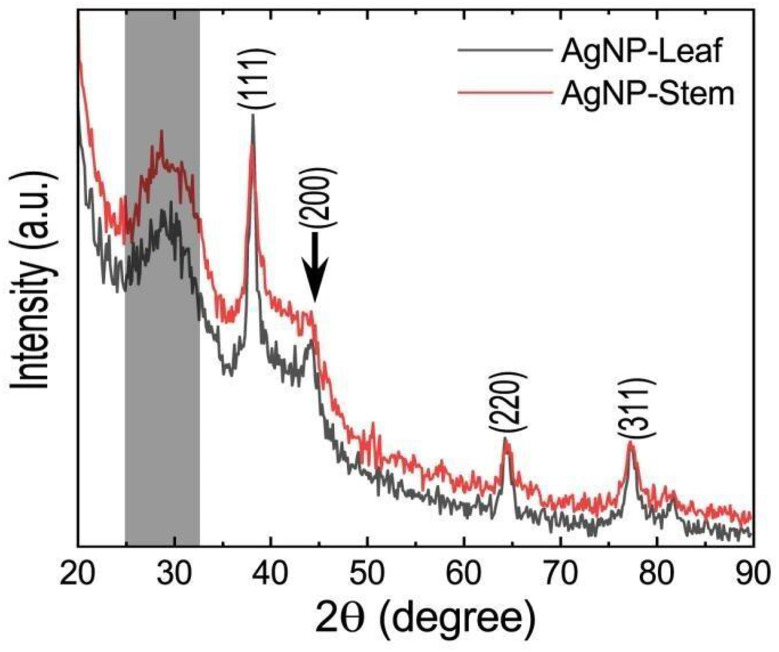
Powder XRD patterns of AgNPs were synthesized using the leaf and stem extracts of *C. asiatica.*

Fig 7 shows XRD patterns of AgNPs synthesized using leaf and stem extracts. Both XRD patterns contain four major diffraction peaks at 2θ = 38.1, 44.1, 64.4, and 77.3º corresponding to (111), (200), (220), and (311) planes of the face-centered cubic crystal structure of silver nanocrystals. The presence of a broad peak in the range of 25° to 32° is attributed to the capping agents from the leaf and stem extracts [51,52]. Nanoparticles were predominantly oriented in the (111) plane, as this plane contained the most intense reflection compared to the other Bragg reflections. Therefore, the crystallite size was also computed using the (111) signal width. The average sizes of nanoparticles calculated using the Debye-Scherrer equation were 9 nm and 8 nm for leaf and stem-assisted synthesized AgNPs, respectively.

**Fig 8:**
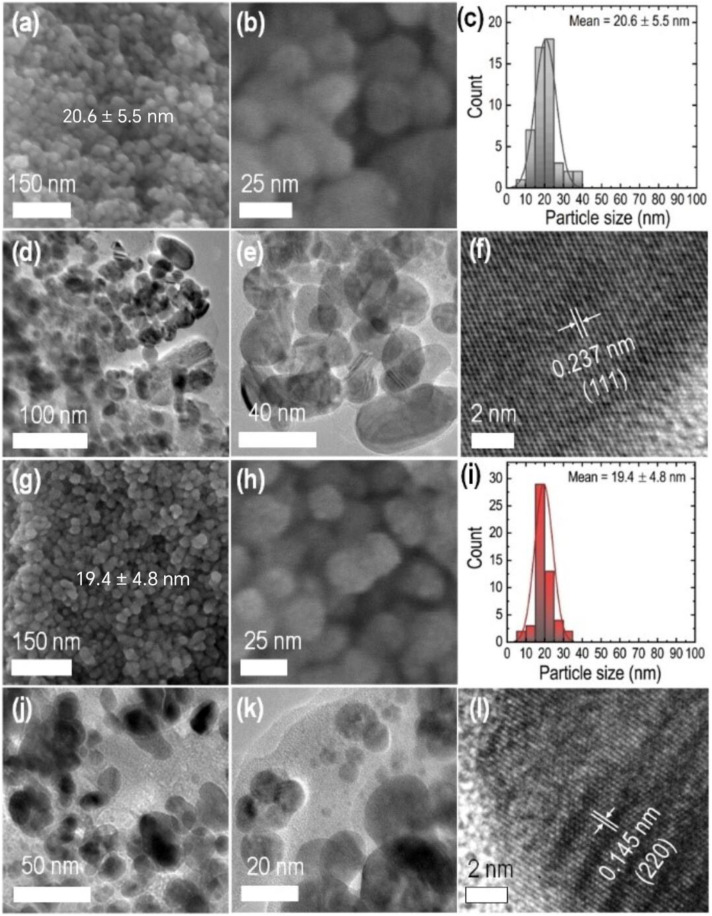
SEM images of leaf extract-synthesized AgNPs at (a) low resolution and (b) high resolution. (c) Histogram showing the size distribution, (d,e) corresponding TEM images, and (f) HR-TEM image showing the crystal lattice of leaf extract-synthesized AgNPs. SEM images of stem extract synthesized AgNPs at (g) low resolution, (h) high resolution, (i) histogram showing the size distribution, (j,k) corresponding TEM images, and (l) HR-TEM image showing the crystal lattice of stem extract synthesized AgNPs.

The surface morphology and structure of the AgNPs were studied using SEM and TEM images (Fig 8). AgNPs synthesized using leaf extract and stem aqueous extract exhibited spherical shapes. The particle size distributions for synthesized silver nanoparticles are provided as histograms. The diameters of nanoparticles synthesized using leaf aqueous extract ranged from 9 to 38 nm with a mean diameter of 20.6 nm. Nanoparticles synthesized using stem extract displayed similar particle size distribution in the range of 8–35 nm with a mean diameter of 19.4 nm. Thus, some anisotropy was noted in the dimensions and configuration of nanoparticles. Similar to our findings, Shrestha et al. have reported a non-uniform size distribution for silver nanoparticles synthesized with aqueous extract of *Polystichum lentum* with an average size of 61.33 nm [53].

**Fig 9:**
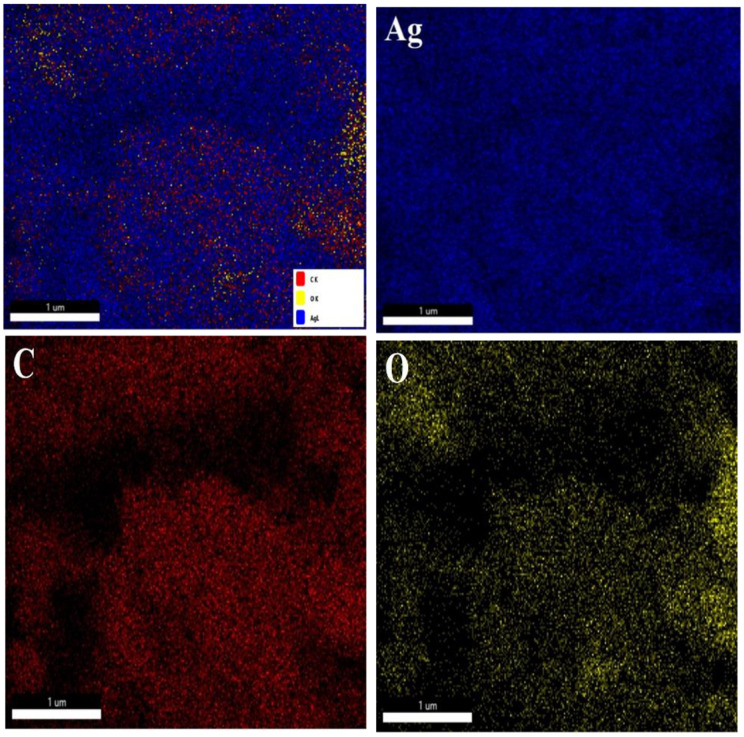
Total elemental mapping and individual color mappings of AgNPs synthesized from leaf aqueous extract.

**Fig 10:**
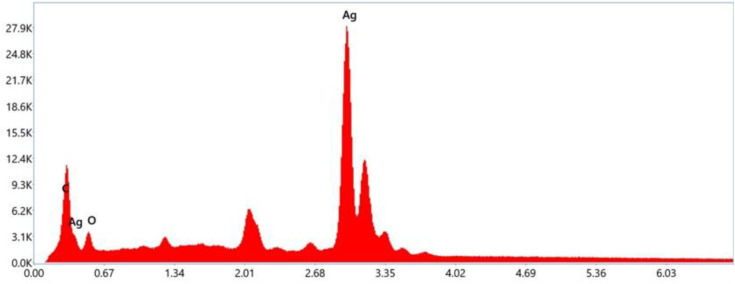
EDX spectrum of synthesized AgNPs from leaf aqueous extract.

The TEM images of AgNPs also reveal spherical shapes and inhomogeneous particle sizes with some aggregates or clusters. The AgNPs were found coated with organic matter (Fig 8e and 8k), probably the stabilizing components of the plant extracts. High-resolution TEM images show lattice fringes of crystalline AgNPs corresponding to FCC crystals. The interlayer d-spacing ca. 0.237 nm (AgNPs synthesized using leaf extract) corresponds to the (111) plane, while the d-spacing ca. 0.145 nm (AgNPs synthesized using stem extract) corresponds to the (220) plane of the FCC crystals of silver. The elemental composition of leaf aqueous extract-synthesized AgNPs was analyzed using EDX spectroscopy. The photographs of elemental mappings are provided in Fig 9. Metallic silver showed a distinct peak at 3 keV in the EDX spectrum (Fig 10). The spectrum also displayed noticeable peaks for carbon and oxygen. These elements represent surface-adsorbed plant secondary metabolites. The weight percentage and atomic percentage of Ag, C, and O are presented in Table 6.

**Table 6:** Elemental composition of AgNPs.

Elements	Weight %	Atomic %
C K	7.4	37.0
O K	3.7	13.8
Ag L	88.9	49.3

### 3.8. Antimicrobial activity of AgNPs

**Fig 11:**
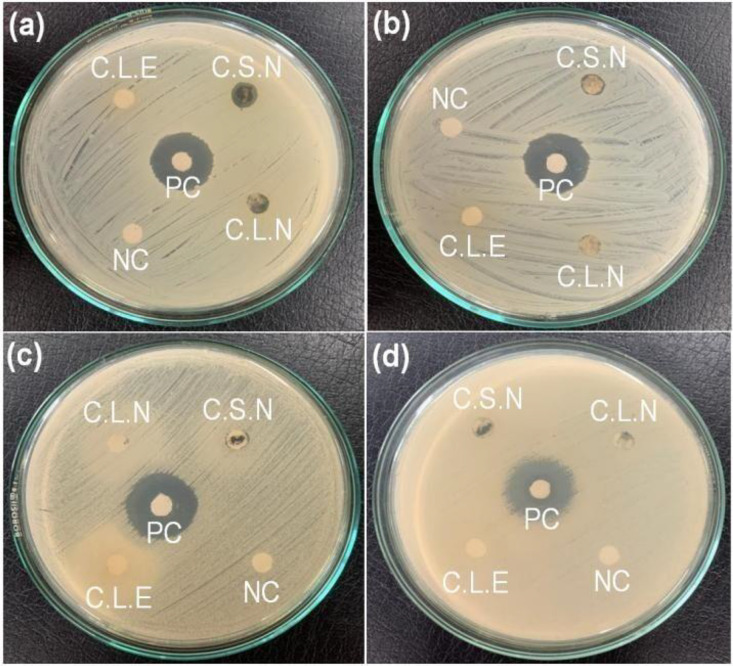
Photographs showing the formation of ZOI against the test bacteria used. (a) *Staphylococcus aureus*, (b) *Shigella sonnei*, (c) *Klebsiella pneumoniae*, and (d) *Escherichia coli.* Here, CLE: *Centella* leaf extract, CLN: *Centella* leaf extract-assisted synthesized AgNPs, CSE: *Centella* stem extract, CSN: *Centella* stem extract-assisted synthesized AgNPs.

Aqueous extracts of *C. asiatica* did not display significant ZOI against test organisms. AgNPs exhibited antibacterial activity against *S. aureus* with ZOI 8 mm and 5 mm, respectively, for stem and leaf-mediated AgNPs (Fig 11 and Table 7). They were inactive against *K. pneumoniae, S. sonnei,* and *E. coli.* The antibacterial activity of silver nanoparticles has been well-reported in the literature. Shrestha et al. have reported ZOIs of 6 mm each for *Shigella sonnei, Staphylococcus aureus,* and *Escherichia coli,* and a ZOI of 7 mm for *Klebsiella pneumoniae. Lallemantia roylena* leaf extract-synthesized Ag nanoparticles have shown excellent activity against *Staphylococcus aureus, Bacillus cereus, Escherichia coli,* and *Shigella flexneri* [54]*.* However, Sandulovici et al. have reported a lack of antibacterial activity for AgNPs against *S. aureus* and *E. coli* at 10 µL*.* ZOIs of 8 and 17.5 mm were observed for 100 µL of AgNPs [55]. Thus, a smaller concentration of AgNPs might have resulted in the absence of ZOIs in the present study. AgNPs bind with bacterial cell membranes and cell walls and cause punctures that lead to leakage of cell content [56]. The minuscule size of nanoparticles allows them to penetrate bacterial membranes. Once inside the bacteria, they can damage vital biomolecules such as DNA, protein, and enzymes [57]. The antibacterial properties of nanoparticles vary with variations in shape, size, and surface charges. Wu et al. have reported an increase in antibacterial activity with a decrease in the size of nanoparticles [58]. Hong et al. have observed higher antibacterial activity for cubic nanoparticles compared to nanospheres and nanowires [59]. Nanoparticles can be combined with available antimicrobial agents to increase their efficacy. In a study by Sharifi-Rad et al., silver chloride nanoparticles combined with amoxicillin showed enhanced antibacterial activity due to a synergistic effect [60].

**Table 7:** ZOI of the tested bacteria against *C. asiatica* extracts and synthesized AgNPs.

Samples	Zones of inhibition (mm)			
	*S. aureus*	*S sonnei*	*K. pneumoniae*	*E. coli*
CLN	5	–	–	–
CSN	8	–	–	–
Neomycin	18	19	18	20

CLN: *Centella* leaf extract-assisted synthesized AgNPs, CSN: *Centella* stem extract-assisted synthesized AgNPs.

## 4. Conclusion

*Centella asiatica* contains alkaloids, flavonoids, saponins, polyphenols, tannins, and glycosides. The high TPC, TFC, and antioxidant activity in the stem and leaf of the plant indicate its high medicinal value. Methanolic and hexane extracts displayed activity against gram-positive and gram-negative bacteria. Aqueous extracts were employed for the green synthesis of silver nanoparticles that also exhibited significant antibacterial activity against *S. aureus*. Thus, the present study identifies *Centella asiatica* and silver nanoparticles as potential sources of antibacterial agents. Additional research to separate, identify, analyze, and characterize the biologically available components from the potent extracts of *C. asiatica* is recommended.

## References

[1] Uprety Y, Asselin H, Boon EK, Yadav S, Shrestha KK. Indigenous use and bio-efficacy of medicinal plants in the Rasuwa District, Central Nepal. J Ethnobiol Ethnomed. 2010;6:3. doi: 10.1186/1746-4269-6-3 20102631 PMC2823594

[2] Gaire BP, Subedi L. Medicinal Plant Diversity and their Pharmacological Aspects of Nepal Himalayas. Pharmacognosy Journal. 2011;3(25):6–17. doi: 10.5530/pj.2011.25.2

[3] Kutal DH, Kunwar RM, Uprety Y, Adhikari YP, Bhattarai S, Adhikari B, et al. Selection of medicinal plants for traditional medicines in Nepal. J Ethnobiol Ethnomed. 2021;17(1):59. doi: 10.1186/s13002-021-00486-5 34656121 PMC8520218

[4] Pandey LK, Sharma KR. Analysis of Phenolic and Flavonoid Content, α-Amylase Inhibitory and Free Radical Scavenging Activities of Some Medicinal Plants. Sci World J. 2022;2022:4000707. doi: 10.1155/2022/4000707 36225946 PMC9550511

[5] Ojima I. Modern natural products chemistry and drug discovery. J Med Chem. 2008;51(9):2587–8. doi: 10.1021/jm701291u 18393400

[6] Numer Al, Sharma KR. Estimation of phenolic content, flavonoid content, antioxidant, and alpha-amylase inhibitory activity of some selected plants from siraha district nepal. Asian J Pharm Clin Res. 2020:18–23. doi: 10.22159/ajpcr.2020.v13i4.36734

[7] Szymanska R, Pospisil P, Kruk J. Corrigendum to “Plant-Derived Antioxidants in Disease Prevention”. Oxid Med Cell Longev. 2017;2017:5092754. doi: 10.1155/2017/5092754 28642809 PMC5470029

[8] Takeuchi Y, Ohkura K, Nishina Y. Self-Assembly Strategies for Graphene Oxide/Silica Nanostructures: Synthesis and Structural Analysis. Bulletin of the Chemical Society of Japan. 2023;96(2):113–9. doi: 10.1246/bcsj.20220279

[9] Ariga K. Nanoarchitectonics: the method for everything in materials science. Bulletin of the Chemical Society of Japan. 2023;97(1). doi: 10.1093/bulcsj/uoad001

[10] Yamaguchi K, Jin X, Yatabe T, Suzuki K. Development of Environmentally Friendly Dehydrogenative Oxidation Reactions Using Multifunctional Heterogeneous Catalysts. Bulletin of the Chemical Society of Japan. 2022;95(9):1332–52. doi: 10.1246/bcsj.20220181

[11] Habeeb Rahuman HB, Dhandapani R, Narayanan S, Palanivel V, Paramasivam R, Subbarayalu R, et al. Medicinal plants mediated the green synthesis of silver nanoparticles and their biomedical applications. IET Nanobiotechnol. 2022;16(4):115–44. doi: 10.1049/nbt2.12078 35426251 PMC9114445

[12] Ahmed RH, Mustafa DE. Green synthesis of silver nanoparticles mediated by traditionally used medicinal plants in Sudan. Int Nano Lett. 2019;10(1):1–14. doi: 10.1007/s40089-019-00291-9

[13] Khanal LN, Sharma KR, Paudyal H, Parajuli K, Dahal B, Ganga GC, et al. Green Synthesis of Silver Nanoparticles from Root Extracts of Rubus ellipticus Sm. and Comparison of Antioxidant and Antibacterial Activity. Journal of Nanomaterials. 2022;2022(1). doi: 10.1155/2022/1832587

[14] Slavin YN, Asnis J, Häfeli UO, Bach H. Metal nanoparticles: understanding the mechanisms behind antibacterial activity. J Nanobiotechnology. 2017;15(1):65. doi: 10.1186/s12951-017-0308-z 28974225 PMC5627441

[15] Sondi I, Salopek-Sondi B. Silver nanoparticles as antimicrobial agent: a case study on E. coli as a model for Gram-negative bacteria. J Colloid Interface Sci. 2004;275(1):177–82. doi: 10.1016/j.jcis.2004.02.012 15158396

[16] Brinkhaus B, Lindner M, Schuppan D, Hahn EG. Chemical, pharmacological and clinical profile of the East Asian medical plant Centella asiatica. Phytomedicine. 2000;7(5):427–48. doi: 10.1016/s0944-7113(00)80065-3 11081995

[17] Biswas D, Mandal S, Chatterjee Saha S, Tudu CK, Nandy S, Batiha GE-S, et al. Ethnobotany, phytochemistry, pharmacology, and toxicity of Centella asiatica (L.) Urban: A comprehensive review. Phytother Res. 2021;35(12):6624–54. doi: 10.1002/ptr.7248 34463404

[18] Sen S, Dutta S. A comprehensive review on Thankuni (Centella asiatica) as an herbal remedy in diabetes mellitus and wound healing. J Pharmacogn Phytochem. 2020;9:1203–9.

[19] Idris FN, Mohd Nadzir M. Comparative Studies on Different Extraction Methods of Centella asiatica and Extracts Bioactive Compounds Effects on Antimicrobial Activities. Antibiotics (Basel). 2021;10(4):457. doi: 10.3390/antibiotics10040457 33920563 PMC8073564

[20] Prakash V, Jaiswal N, Srivastava M. A review on medicinal properties of centella asiatica. Asian J Pharm Clin Res. 2017;10(10):69. doi: 10.22159/ajpcr.2017.v10i10.20760

[21] Legiawati L, Bramono K, Indriatmi W, Yunir E, Setiati S, Jusman SWA, et al. Oral and Topical Centella asiatica in Type 2 Diabetes Mellitus Patients with Dry Skin: A Three-Arm Prospective Randomized Double-Blind Controlled Trial. Evid Based Complement Alternat Med. 2020;2020:7253560. doi: 10.1155/2020/7253560 32908567 PMC7471832

[22] Jahan R, Hossain S, Seraj S, Nasrin D, Khatun Z, Das PR, et al. Centella asiatica (L.) Urb.: Ethnomedicinal uses and their scientific validations. American-Eurasian J Sustain Agric. 2012;6(4):261–70.

[23] Kunwar RM, Adhikari N. Ethnomedicine of Dolpa district, Nepal: the plants, their vernacular names and uses. Iyonia. 2005;8:43–9.

[24] Balami NP. Ethnomedicinal uses of plants among the Newar community of Pharping village of Kathmandu District, Nepal. Tribhuvan University Journal. 1970;24(1):13–9. doi: 10.3126/tuj.v24i1.251

[25] Barchan A, Bakkali M, Arakrak A, Pagan R, Laglaoui A. The effects of solvent polarity on the phenolic contents and antioxidant activity of three Mentha species extracts. Int J Curr Microbial App Sci. 2014;3(11):399–412.

[26] Rekha B, Seema K, Tapan D. Phytochemical profiling, assessment of total phenolic content, total flavonoid content, and antioxidant activity of ethnomedicinal plant, meyna spinosa from assam. Asian J Pharm Clin Res. 2019;:61–3. doi: 10.22159/ajpcr.2019.v12i11.34616

[27] Ainsworth EA, Gillespie KM. Estimation of total phenolic content and other oxidation substrates in plant tissues using Folin-Ciocalteu reagent. Nat Protoc. 2007;2(4):875–7. doi: 10.1038/nprot.2007.102 17446889

[28] Chang C-C, Yang M-H, Wen H-M, Chern J-C. Estimation of total flavonoid content in propolis by two complementary colometric methods. Journal of Food and Drug Analysis. 2020;10(3). doi: 10.38212/2224-6614.2748

[29] Alabri THA, Musalami ASHA, Hossain MA, Weli AM, Riyami QA. Comparative study of phytochemical screening, antioxidant and antimicrobial capacities of fresh and dry leaves crude plant extracts of Datura metel L. J King Saud Univ Sci. 2014;26:237–43.

[30] Holder IA, Boyce ST. Agar well diffusion assay testing of bacterial susceptibility to various antimicrobials in concentrations non-toxic for human cells in culture. Burns. 1994;20(5):426–9. doi: 10.1016/0305-4179(94)90035-3 7999271

[31] Wayne PA. CLSI. Performance standards for antimicrobial susceptibility testing. In: 30th ed. CLSI Supplement M100. Clinical and Laboratory Standards Institute, 2020.

[32] Jalab J, Abdelwahed W, Kitaz A, Al-Kayali R. Green synthesis of silver nanoparticles using aqueous extract of Acacia cyanophylla and its antibacterial activity. Heliyon. 2021;7(9):e08033. doi: 10.1016/j.heliyon.2021.e08033 34611564 PMC8477989

[33] Sieberi BM, Omwenga GI, Wambua RK, Samoei JC, Ngugi MP. Screening of the Dichloromethane: Methanolic Extract of Centella asiatica for Antibacterial Activities against Salmonella typhi, Escherichia coli, Shigella sonnei, Bacillus subtilis, and Staphylococcus aureus. ScientificWorldJournal. 2020;2020:6378712. doi: 10.1155/2020/6378712 32694956 PMC7350070

[34] Saranya S, Nair AV, Prathapan M, Neethu S, Kumar NS. Phytochemical analysis of Centella asiatica L. leaf extracts. Int J Approx Reason. 2017;5:1828–32.

[35] Khadka A, Budha Magar A, Sharma KR. Chemical Profiling and Biological Activities on Nepalese Medicinal Plant Extracts and Isolation of Active Fraction of Nyctanthes arbor-tristis. Sci World Jl. 2024;2024:5080176. doi: 10.1155/2024/5080176 38515931 PMC10957254

[36] Ovais M, Khalil AT, Islam NU, Ahmad I, Ayaz M, Saravanan M, et al. Role of plant phytochemicals and microbial enzymes in biosynthesis of metallic nanoparticles. Appl Microbiol Biotechnol. 2018;102(16):6799–814. doi: 10.1007/s00253-018-9146-7 29882162

[37] Sharifi-Rad M, Mohanta YK, Pohl P, Jaradat N, Aboul-Soud MAM, Zengin G. Variation of phytochemical constituents, antioxidant, antibacterial, antifungal, and anti-inflammatory properties of Grantia aucheri (Boiss.) at different growth stages. Microb Pathog. 2022;172:105805. doi: 10.1016/j.micpath.2022.105805 36179974

[38] Park K. The Role of Dietary Phytochemicals: Evidence from Epidemiological Studies. Nutrients. 2023;15(6):1371. doi: 10.3390/nu15061371 36986101 PMC10054640

[39] Catalano E. Role of phytochemicals in the chemoprevention of tumors. arXiv. 2016:1605.04519. doi: 10.48550/arXiv.1605.04519

[40] Carvalho MTB, Araújo-Filho HG, Barreto AS, Quintans-Júnior LJ, Quintans JSS, Barreto RSS. Wound healing properties of flavonoids: A systematic review highlighting the mechanisms of action. Phytomedicine. 2021;90:153636. doi: 10.1016/j.phymed.2021.153636 34333340

[41] Benmohamed M, Guenane H, Messaoudi M, Zahnit W, Egbuna C, Sharifi-Rad M, et al. Mineral Profile, Antioxidant, Anti-Inflammatory, Antibacterial, Anti-Urease and Anti-α-Amylase Activities of the Unripe Fruit Extracts of Pistacia atlantica. Molecules. 2023;28(1):349. doi: 10.3390/molecules28010349 36615545 PMC9824078

[42] Bhusal M, Sharma K, Magar AB, Pant J, Sharma KR. Chemical analysis and biological activities on solvent extracts from different parts of Rhus chinensis mill. Nat Prod Res. 2024:1–7. doi: 10.1080/14786419.2024.2387831 39094015

[43] Rashid MdH-O-, Akter MstM, Uddin J, Islam S, Rahman M, Jahan K, et al. Antioxidant, cytotoxic, antibacterial and thrombolytic activities of Centella asiatica L.: possible role of phenolics and flavonoids. Clin Phytosci. 2023;9(1). doi: 10.1186/s40816-023-00353-8

[44] Tungmunnithum D, Thongboonyou A, Pholboon A, Yangsabai A. Flavonoids and Other Phenolic Compounds from Medicinal Plants for Pharmaceutical and Medical Aspects: An Overview. Medicines (Basel). 2018;5(3):93. doi: 10.3390/medicines5030093 30149600 PMC6165118

[45] Preiser J-C. Oxidative stress. JPEN J Parenter Enteral Nutr. 2012;36(2):147–54. doi: 10.1177/0148607111434963 22301329

[46] Ondeko DA, Juma BF, Baraza LD, Nyongesa PK. LC-ESI/MS and GC-MS Methanol Extract Analysis, Phytochemical and Antimicrobial Activity Studies of Centella asiatica. AJOCS. 2020:32–51. doi: 10.9734/ajocs/2020/v8i319046

[47] Shrestha DK, Jaishi DR, Ojha I, Ojha DR, Pathak I, Magar AB, et al. Plant assisted synthesis of silver nanoparticles using Persicaria perfoliata (L.) for antioxidant, antibacterial, and anticancer properties. Heliyon. 2024;10(23):e40543. doi: 10.1016/j.heliyon.2024.e40543 39660180 PMC11629186

[48] Dangi S, Gupta A, Gupta DK, Singh S, Parajuli N. Green synthesis of silver nanoparticles using aqueous root extract of Berberis asiatica and evaluation of their antibacterial activity. Chemical Data Collections. 2020;28:100411. doi: 10.1016/j.cdc.2020.100411

[49] Javed R, Ijaz S, Hameed H, Nazish M, Sharif MS, Afreen A, et al. Phytochemical-Mediated Biosynthesis of Silver Nanoparticles from Strobilanthes glutinosus: Exploring Biological Applications. Micromachines (Basel). 2023;14(7):1372. doi: 10.3390/mi14071372 37512683 PMC10386440

[50] Fard SE, Tafvizi F, Torbati MB. Silver nanoparticles biosynthesised using Centella asiatica leaf extract: apoptosis induction in MCF-7 breast cancer cell line. IET Nanobiotechnol. 2018;12(7):994–1002. doi: 10.1049/iet-nbt.2018.5069 30247143 PMC8676233

[51] Ibrahim HMM. Green synthesis and characterization of silver nanoparticles using banana peel extract and their antimicrobial activity against representative microorganisms. Journal of Radiation Research and Applied Sciences. 2015;8(3):265–75. doi: 10.1016/j.jrras.2015.01.007

[52] Suresh G, Gunasekar PH, Kokila D, Prabhu D, Dinesh D, Ravichandran N, et al. Green synthesis of silver nanoparticles using Delphinium denudatum root extract exhibits antibacterial and mosquito larvicidal activities. Spectrochim Acta A Mol Biomol Spectrosc. 2014;127:61–6. doi: 10.1016/j.saa.2014.02.030 24632157

[53] Shrestha DK, Magar AB, Bhusal M, Baraili R, Pathak I, Joshi PR, et al. Synthesis of Silver and Zinc Oxide Nanoparticles Using Polystichum lentum Extract for the Potential Antibacterial, Antioxidant, and Anticancer Activities. Journal of Chemistry. 2024;2024(1). doi: 10.1155/2024/1876560

[54] Sharifi-Rad M, Elshafie HS, Pohl P. Green synthesis of silver nanoparticles (AgNPs) by Lallemantia royleana leaf Extract: Their Bio-Pharmaceutical and catalytic properties. Journal of Photochemistry and Photobiology A: Chemistry. 2024;448:115318. doi: 10.1016/j.jphotochem.2023.115318

[55] Sandulovici RC, Carmen-Marinela M, Grigoroiu A, Moldovan CA, Savin M, Ordeanu V, et al. The Physicochemical and Antimicrobial Properties of Silver/Gold Nanoparticles Obtained by “Green Synthesis” from Willow Bark and Their Formulations as Potential Innovative Pharmaceutical Substances. Pharmaceuticals (Basel). 2022;16(1):48. doi: 10.3390/ph16010048 36678545 PMC9867178

[56] Tiwari V, Mishra N, Gadani K, Solanki PS, Shah NA, Tiwari M. Mechanism of Anti-bacterial Activity of Zinc Oxide Nanoparticle Against Carbapenem-Resistant Acinetobacter baumannii. Front Microbiol. 2018;9:1218. doi: 10.3389/fmicb.2018.01218 29928271 PMC5997932

[57] Thakral F, Bhatia GK, Tuli HS, Sharma AK, Sood S. Zinc Oxide Nanoparticles: from Biosynthesis, Characterization, and Optimization to Synergistic Antibacterial Potential. Curr Pharmacol Rep. 2021;7(1):15–25. doi: 10.1007/s40495-021-00248-7

[58] Wu Y, Yang Y, Zhang Z, Wang Z, Zhao Y, Sun L. A facile method to prepare size-tunable silver nanoparticles and its antibacterial mechanism. Advanced Powder Technology. 2018;29(2):407–15. doi: 10.1016/j.apt.2017.11.028

[59] Hong X, Wen J, Xiong X, Hu Y. Shape effect on the antibacterial activity of silver nanoparticles synthesized via a microwave-assisted method. Environ Sci Pollut Res Int. 2016;23(5):4489–97. doi: 10.1007/s11356-015-5668-z 26511259

[60] Sharifi-Rad M, Pohl P. Synthesis of Biogenic Silver Nanoparticles (AgCl-NPs) Using a Pulicaria vulgaris Gaertn. Aerial Part Extract and Their Application as Antibacterial, Antifungal and Antioxidant Agents. Nanomaterials (Basel). 2020;10(4):638. doi: 10.3390/nano10040638 32235379 PMC7221712

